# Role of Macrophages in Early Host Resistance to Respiratory *Acinetobacter baumannii* Infection

**DOI:** 10.1371/journal.pone.0040019

**Published:** 2012-06-29

**Authors:** Hongyu Qiu, Rhonda KuoLee, Greg Harris, Nico Van Rooijen, Girishchandra B. Patel, Wangxue Chen

**Affiliations:** 1 Institute for Biological Sciences, National Research Council Canada, Ottawa, Ontario, Canada; 2 Department of Molecular Cell Biology, Vrije Universiteit Medical Center, Amsterdam, The Netherlands; 3 Department of Biology, Brock University, St. Catharines, Ontario, Canada; Monash University, Australia

## Abstract

*Acinetobacter baumannii* is an emerging bacterial pathogen that causes nosocomial pneumonia and other infections. Although it is recognized as an increasing threat to immunocompromised patients, the mechanism of host defense against *A. baumannii* infection remains poorly understood. In this study, we examined the potential role of macrophages in host defense against *A. baumannii* infection using *in vitro* macrophage culture and the mouse model of intranasal (i.n.) infection. Large numbers of *A. baumannii* were taken up by alveolar macrophages *in vivo* as early as 4 h after i.n. inoculation. By 24 h, the infection induced significant recruitment and activation (enhanced expression of CD80, CD86 and MHC-II) of macrophages into bronchoalveolar spaces. *In vitro* cell culture studies showed that *A. baumannii* were phagocytosed by J774A.1 (J774) macrophage-like cells within 10 minutes of co-incubation, and this uptake was microfilament- and microtubule-dependent. Moreover, the viability of phagocytosed bacteria dropped significantly between 24 and 48 h after co-incubation. Infection of J774 cells by *A. baumannii* resulted in the production of large amounts of proinflammatory cytokines and chemokines, and moderate amounts of nitric oxide (NO). Prior treatment of J774 cells with NO inhibitors significantly suppressed their bactericidal efficacy (P<0.05). Most importantly, *in vivo* depletion of alveolar macrophages significantly enhanced the susceptibility of mice to i.n. *A. baumannii* challenge (P<0.01). These results indicate that macrophages may play an important role in early host defense against *A. baumannii* infection through the efficient phagocytosis and killing of *A. baumannii* to limit initial pathogen replication and the secretion of proinflammatory cytokines and chemokines for the rapid recruitment of other innate immune cells such as neutrophils.

## Introduction

*Acinetobacter baumannii* is a ubiquitous, Gram-negative, opportunistic pathogen that frequently induces nosocomial and community-acquired pneumonia, skin and urinary tract infections, and bacteremia [1]–[3], especially in immunocompromised individuals [4]. Moreover, *A. baumannii* infections are becoming increasingly difficult to treat due to the rapid development of resistance to antibiotics [3], [5]. Thus, *A. baumannii* infection can lead to significant morbidity and mortality, with an overall 30-day mortality rate as high as 49% for respiratory tract infections [6].

Despite its clinical importance, relatively little is known about the innate host defense mechanisms against respiratory *A. baumannii* infection. Recent studies by several groups, including us, have shown that CD14, TLR-4 signaling, neutrophils, NADPH phagocyte oxidase, and complement are crucial in the control of local bacterial multiplication and subsequent extrapulmonary dissemination [7]–[12]. On the other hand, TLR-2, NOS2 or IL-17 play little to no role [9], [11], [13].

Similar to the neutrophil, the macrophage is another important phagocyte that is generally involved in host defense against pathogen invasion. Alveolar macrophages (AMs) are the first line of innate immune cells in the distal respiratory tract that are capable of detecting and eliminating invading pathogens as well as initiating the early host immune response. In this regard, AMs play a critical role in host resistance against both intracellular and extracellular bacterial pathogens [14]–[17], and are capable of clearing a low inoculum of bacteria without the recruitment of neutrophils [18]. However, to the best of our knowledge, there are no studies that have systemically evaluated the macrophage function during respiratory *A. baumannii* infection. In this study, we examined the relative contribution of macrophages in the host defense against *A. baumannii* infection using *in vitro* J774A.1 (J774) macrophage cell culture and the mouse model of intranasal (i.n.) *A. baumannii* infection. Our data suggest that macrophages may play an important role in the early host defense against respiratory *A. baumannii* infection.

## Results and Discussion

### Alveolar macrophage responses to intranasal *A. baumannii* infection in mice

**Figure 1:**
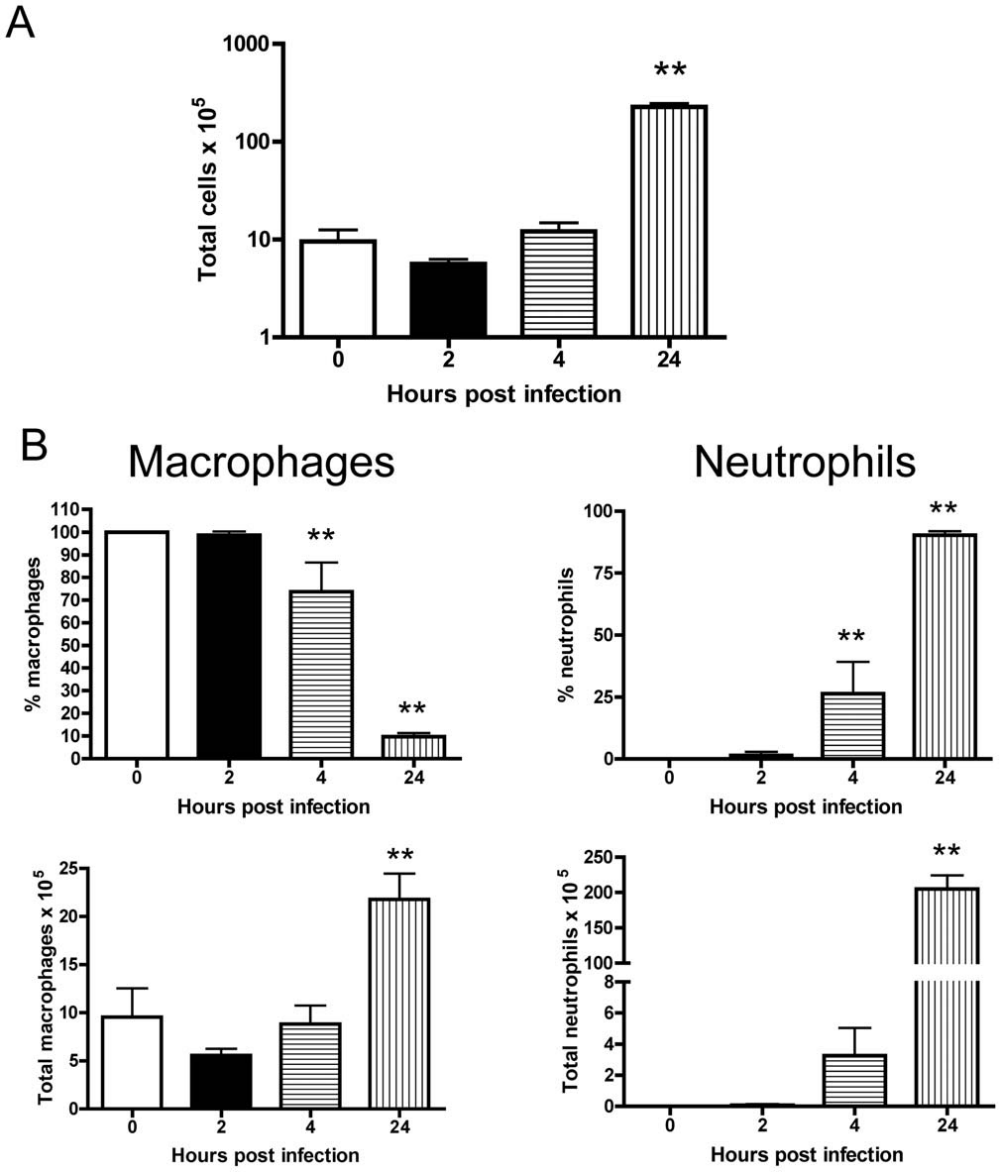
Brochoalveolar macrophage responses to intranasal inoculation with *A. baumannii*. Groups of C57BL/6 mice (n = 5 per group) were i.n. inoculated with 10^8^ CFU of freshly cultured *A. baumannii*. The mice were euthanized at the indicated times and the lungs were lavaged. The total number of bronchoalveolar lavage (BAL) fluid cells was determined by a hemocytometer (A) and the differential count of macrophages (B, left) and neutrophils (B, right) was determined by examining Hema3-stained cytospin slides. Data are representative of three independent experiments with similar results and presented as mean ± SD. **P<0.01 vs 0 h.

Since AMs are the front line of innate immune cells that combat respiratory pathogens, we first determined the kinetics of AM recruitment in C57BL/6 mice in response to an i.n. *A. baumannii* infection. As shown in Fig. 1A, the total number of bronchoalveolar lavage (BAL) cells was moderately reduced at 2 hours post infection (hpi) with approximately 10^8^ colony-forming units (CFU) *A. baumannii*, but was significantly increased by 24 hpi (P<0.01). Macrophages comprised nearly all of the cells recovered from the BAL fluid at 0 and 2 (100% and 98.7%, respectively) hpi. By 4 hpi, some neutrophils (25%) were present in the BAL and by 24 hpi, approximately 90% of the all BAL cells were neutrophils (Fig. 1B). Despite the decrease in the proportion of macrophages in the BAL fluid at 24 hpi, the absolute number of macrophages actually increased significantly at this time point (P<0.01)(Fig. 1B).

**Figure 2:**
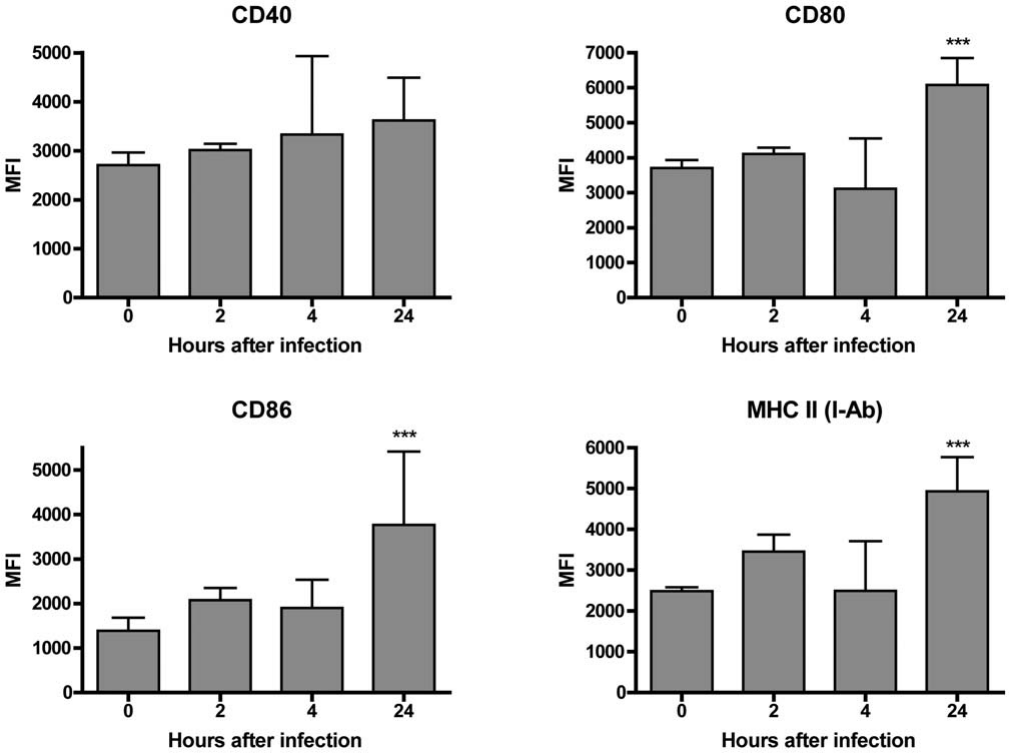
Activation of CD11c+ alveolar macrophages following intranasal inoculation of *A. baumannii.* Groups of C57BL/6 mice were i.n. inoculated with 10^8^ CFU of freshly cultured *A. baumannii*. The mice were euthanized at the indicated times and the lungs were lavaged. The expression of CD40, CD80, CD86 and MHCII by CD11c+ macrophages, measured as mean fluorescence intensity (MFI), was determined by FACS analysis. Data are compiled from two independent experiments with similar results, and are presented as mean ± SD (n = 6 per time point). ***P<0.001 vs 0 h.

**Figure 3:**
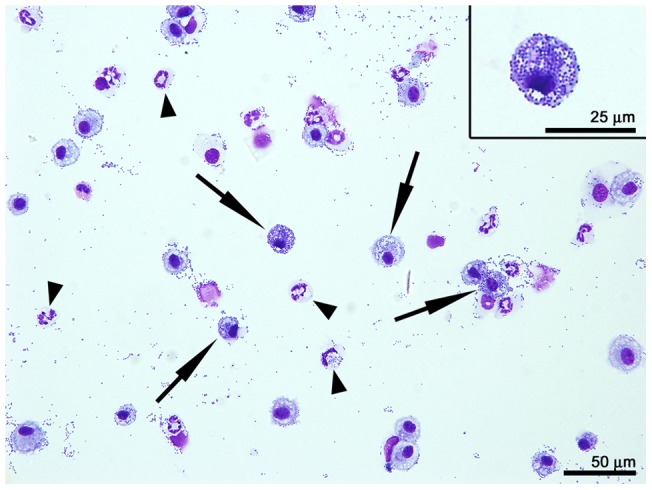
*In vivo* phagocytosis of *A. baumannii* by alveolar macrophages. C57BL/6 mice were i.n. inoculated with 10^8^ CFU of freshly cultured *A. baumannii* and euthanized 4 h later. The lungs were lavaged and the lavage fluid was used for the preparation of cytospin slides. BAL cells were stained with Hema3 and observed under a light microscope. A representative photo of at least 10 samples is presented. Arrows: alveolar macrophages containing large numbers of *A. baumannii*. Arrowheads: neutrophils containing small numbers of *A. baumannii*.

Flow cytometric analysis of macrophage surface markers showed no substantial changes in the activation markers CD40, CD80, CD86 or MHC-II (I-A^b^) at 2 or 4 hpi, but the expression of CD80, CD86 and MHC-II was significantly increased at 24 hpi (P<0.001)(Fig. 2). More importantly, many AMs at 4 hpi contained high numbers of bacteria within their cytoplasm (Fig. 3) while only a few bacteria were visible in the cytoplasm of small numbers of neutrophils at this time point. These results suggest that *A. baumannii* induce moderate activation and recruitment of AMs into the lungs, and AMs are capable of taking up *A. baumannii* cells shortly after i.n. infection of the mice.

### *In vitro* uptake of *A. baumannii* by macrophages

**Figure 4:**
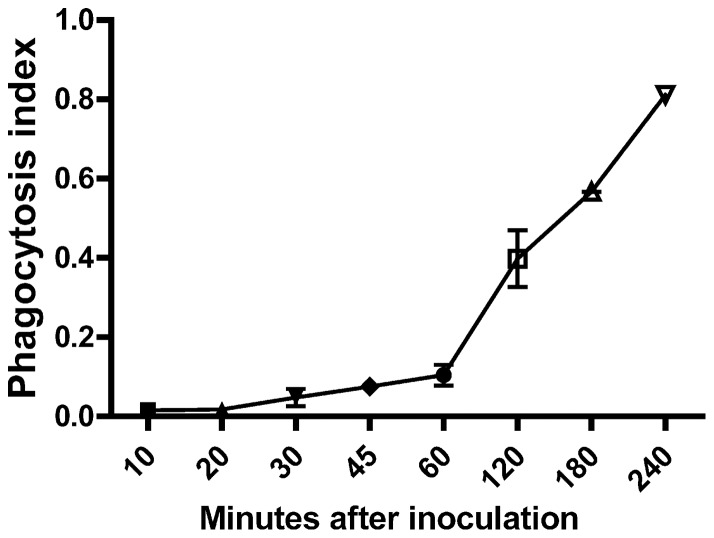
Time dependent *in vitro* phagocytosis of *A. baumannii* by J774 macrophages. J774 (5×10^5^ cells) cells were infected with 100 MOI of *A. baumannii* in 500 μl DMEM complete medium for the indicated time. The cells were then treated with 100 μg/ml gentamicin for 2 h and lysed as described in Materials and Methods. The released ingested bacteria were enumerated by serial dilution on BHI agar supplemented with 50 μg/ml streptomycin. The phagocytosis index was calculated by using the following formula: [bacteria recovered (CFU/ml)/bacteria inoculated (CFU/ml)]×100 = % phagocytosed. Data are compiled from three separate experiments and are expressed as means of individual experiments ± SD where applicable (30, 60 and 120 min).

To further characterize the interaction between *A. baumannii* and macrophages, we examined the *in vitro* uptake of *A. baumannii* by the murine macrophage cell line J774A.1 (J774 cells). *A. baumannii* were incubated with J774 cells at a multiplicity of infection (MOI) dose of 100. After 4 h incubation, 1.36±0.13×10^6^ CFU bacteria were detected inside J774 macrophages, representing about 3% of the total initial inoculated bacteria. Moreover, the uptake of *A. baumannii* by J774 macrophages was time-dependent. The bacteria were internalized by the macrophages as early as 10 min after inoculation, and the level of uptake continued to increase until the end of the treatment (4 h)(Fig. 4). These results support the finding of the above *in vivo* study and showed that macrophages can rapidly and efficiently phagocytose *A. baumannii in vitro* in a time-dependent fashion without the presence of antibody or complement opsonization.

### Cytokine/chemokine responses in *A. baumannii-*infected J774 macrophages

**Figure 5:**
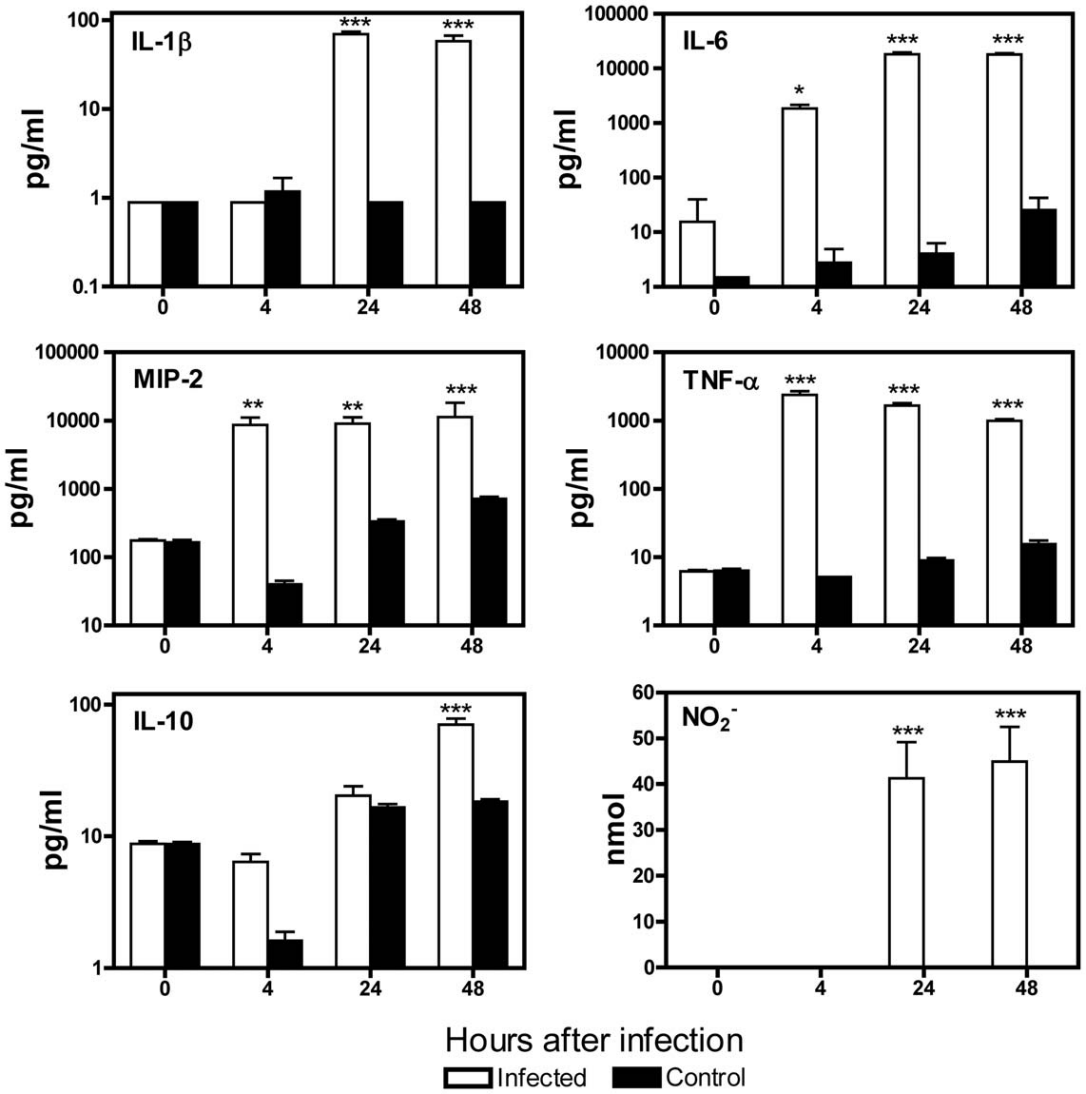
Proinflammatory cytokine and chemokine production by *A. baumannii-*infected J774 macrophages. J774 cells were infected with *A. baumannii* (MOI = 100) for 2 h. The cells were then rinsed with HBSS and treated for 2 h with complete medium containing 100 μg/ml gentamicin to kill extracellular bacteria. Cell culture supernatants were collected at 0 (no infection), 4, 24 and 48 h after bacterial inoculation. The concentrations of indicated cytokines/chemokines were measured by Luminex and the NO level was assessed using Griess reagent. Data are presented as mean ± SD (n = 3). **P<0.01, ***P<0.001 vs 0 h.

To further understand the interaction between *A. baumannii* and macrophages, we determined the levels of interleukin (IL)-1β, IL-6, macrophage inflammatory protein (MIP)-2, tumor necrosis factor (TNF)-α, IL-10 and nitric oxide (NO) in the culture supernatant of J774 cells at 0 (no infection), 4, 24 or 48 h after inoculation with 100 MOI of *A. baumannii* (Fig. 5). This panel of cytokines and chemokines was selected for study because they have previously been implicated in the immunopathogenesis of *A. baumannii* infection in mouse models and in human cell culture studies [7], [9], [11], [13], [19]–[21]. At 4 h, the levels of IL-6 and TNF-α increased >100-fold (P<0.05) and >400-fold (P<0.001), respectively, as compared to 0 h. Similarly, the level of MIP-2 also increased about 100-fold (P<0.001) although there was no significant change in the levels of IL-1β or IL-10. At 24 and 48 h, levels of MIP-2 and TNF-α were maintained at the levels observed at 4 h while IL-6 and IL-1β concentrations increased significantly and peaked at 24 h (P<0.01)(Fig. 5). The IL-10 level did not change significantly in the first 24 h but then increased significantly at 48 h, in comparison with uninfected cultures (P<0.001). Although NO was undetectable (<2.6 nmol) at 0 or 4 h, significant amounts of NO were detected in infected culture supernatants collected 24 and 48 hpi (P<0.001)(Fig. 5).

Our previous studies have shown that respiratory *A. baumannii* infection in mice induces local production of moderate amounts of inflammatory cytokines TNF-α, IL-1, IL-6, MIP-2, and anti-inflammatory cytokine IL-10 [7]. However, the cellular sources of these cytokines were not identified. Here we show that macrophages, after infection with *A. baumannii*, produced high levels of IL-6, TNF-α and MIP-2 as early as 4 hpi. MIP-2 and TNF-α levels peaked at 4 hpi, and IL-6 and IL-1β levels peaked at 24 hpi, respectively (Fig. 5). These results suggest that macrophages participate in the early inflammatory responses and host defense against *A. baumannii* infection. In this regard, it is interesting to note that the production of MIP-2 (a key neutrophil chemotactic chemokine in mice) by macrophages rose rapidly and reached a high level 4 h after infection. It is possible that macrophage-derived MIP-2 may contribute significantly to the early neutrophil recruitment following *A. baumannii* infection *in vivo* but this can only be definitively concluded after conducting studies using MIP-2 knockout mice.

### Mechanisms of *A. baumannii* entry into macrophages

**Figure 6:**
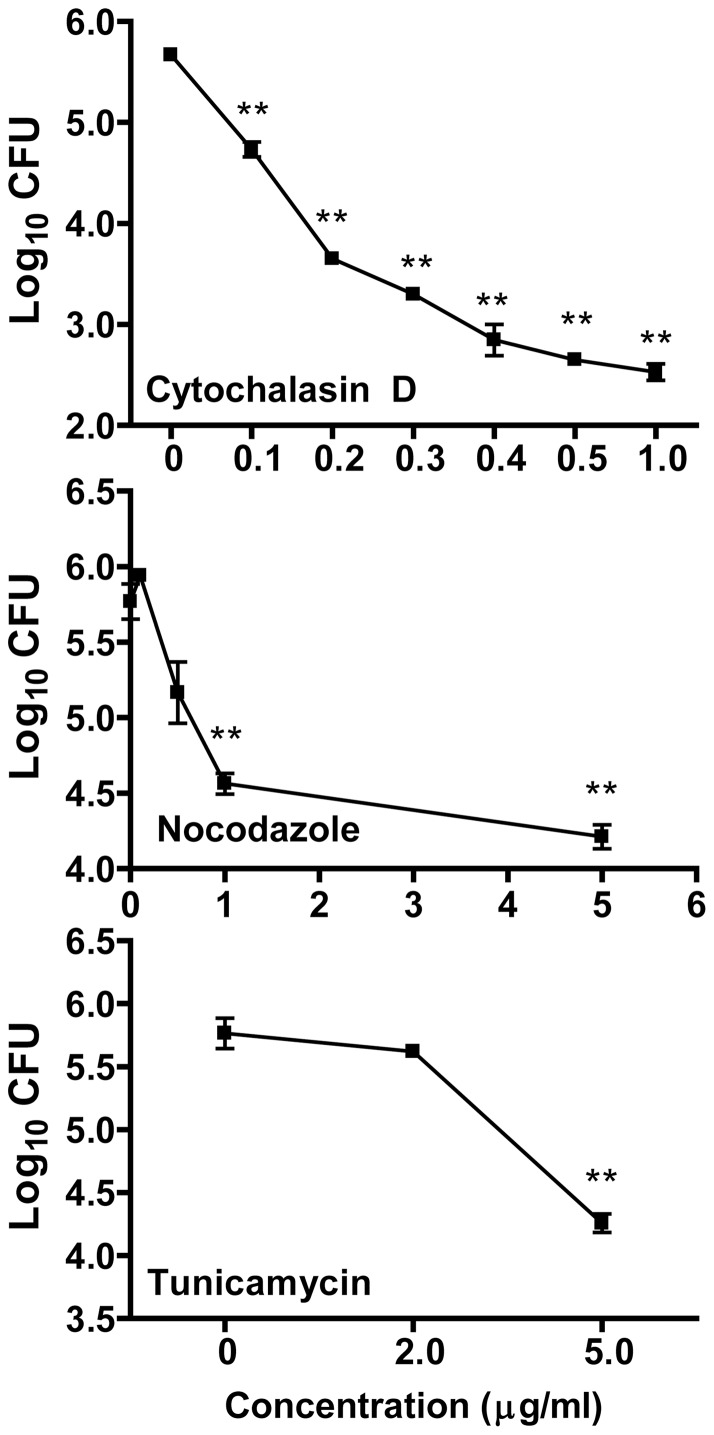
Effect of biochemical inhibitors on the uptake of *A. baumannii* by J774 cells. J774 cells were pretreated for 1 h with various concentrations of the indicated biochemical inhibitors. The cells were then infected with 100 MOI of *A. baumannii* for 2 h, followed by a 2 h incubation with gentamicin to kill extracellular bacteria. The numbers of phagocytosed bacteria were determined as detailed in Figure 4. Data are presented as mean ± SD (n = 3). **P<0.01, vs 0 h.

It has been well established that entry of bacteria into macrophages depends on different receptors, and requires certain cell skeleton changes. To elucidate the potential molecular mechanisms involved in the uptake of *A. baumannii* by macrophages, we examined the effect of cytochalasin D, nocodazole, and tunicamycin on the uptake of *A. baumannii* by J774 cells. Cytochalasin D interferes with the microfilament system rearrangement of eukaryotic cells by blocking actin polymerization, which is necessary for macrophage phagocytosis [22]–[23]. Nocodazole specifically disrupts the cellular microtubulin system, which is essential for receptor-mediated endocytosis of small particles [23]–[24] while tunicamycin interferes with surface receptor N-glycosylation [20]. As shown in Fig. 6, pretreatment of J774 macrophages with cytochalasin D inhibited the uptake of *A. baumannii* in a dose-dependent manner, with >80% of inhibition at a concentration as low as 0.1 μg/ml. The uptake of *A. baumannii* by J774 macrophages was also inhibited by nocodazole and tunicamycin (Fig. 6). These results indicate that the phagocytosis of *A. baumannii* by J774 macrophages requires intact/functional microfilament and microtubulin systems.

### Killing of phagocytosed *A. baumannii* by J774 macrophages

**Figure 7:**
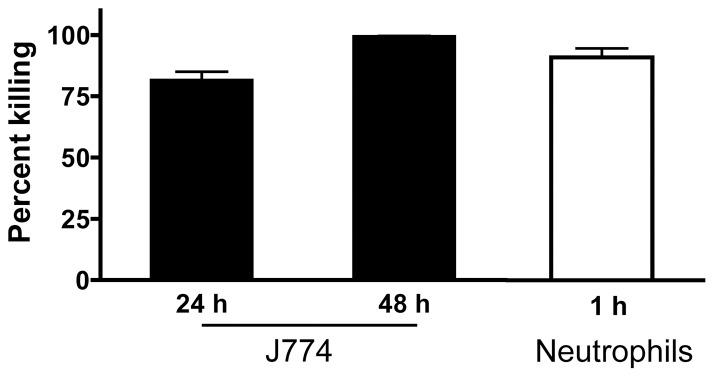
Survival of ingested *A. baumannii* in J774 cells and neutrophils. J774 cells and neutrophils were infected with 100 or 10 MOI of *A. baumannii*, respectively, and extracellular bacteria were killed by gentamycin as detailed in Figure 4. The cells were lysed at indicated times (hours) after infection to determine viable bacterial counts by quantitative bacteriology. Data are expressed as the percentage of the ingested bacteria that were killed by J774 cells or neutrophils at indicated time and presented as mean ± SD (n = 3 for J774 macrophages and n = 9 for neutrophils). The neutrophil data are compiled from three independent experiments.

The killing of phagocytosed pathogens is an important function of macrophages, and is also a major host defense strategy in combating extracellular bacterial infections. We therefore determined the ability of J774 macrophages to kill phagocytosed *A. baumannii*. J774 cells were infected for 2 h with 100 MOI of *A. baumannii*, and the cells were lysed after 24 or 48 h to recover viable *A. baumannii*. As shown in Fig. 7, more than 80% of J774 phagocytosed *A. baumannii* cells were killed at 24 h post-phagocytosis, and 99% were killed by 48 h. However, >90% of *A. baumannii* phagocytosed by mouse neutrophils were killed after only one hour of incubation (Fig. 7). Thus, it would appear that in comparison to neutrophils, macrophages are less efficient in killing phagocytosed *A. baumannii*.

### Potential mechanism of macrophage-mediated killing of *A. baumannii*

**Figure 8:**
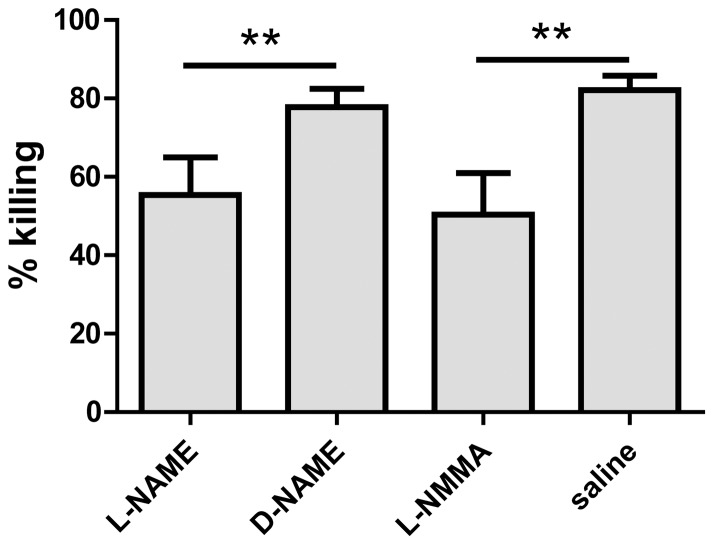
Effect of iNOS inhibitors on the killing of *A. baumannii* by macrophages. J774 cells were treated with 2 mM iNOS inhibitors (L-NAME or L-NMMA) or controls (D-NAME or media) before being infected with *A. baumannii* as described in Figure 4. Cells were lysed after 24 h and the numbers of viable *A. baumannii* were determined by plating on BHI-S plates. The inhibition of bacterial growth is calculated by the formula: % inhibition = 100 – [(viable bacteria at 24 h/viable bacteria at 4 h)×100]. Data are presented as mean ± SD (n = 3) and are representative of three independent experiments. **P< 0.01 vs respective controls.

Macrophages employ a variety of mediators such as NO, reactive oxygen species and phagolysosome-associated enzymes to kill phagocytosed pathogens [18], [25]. Since NO is one of the major effector molecules in bactericidal action of macrophages [26] and NO production is significantly increased in *A. baumannii*-infected macrophages (Fig. 5), we examined the potential significance of NO in macrophage killing of *A. baumannii*. J774 cells were treated with NO inhibitor L-NAME or its non-functional isomer D-NAME one hour before being infected with *A. baumannii*. As shown in Fig. 8, both L-NAME and D-NAME treated macrophages killed substantial numbers of internalized bacteria at 24 hpi. However, the inhibition of *A. baumannii* in L-NAME-treated J774 cells was ∼20% greater than in the D-NAME-treated cells (P<0.01), indicating a role for NO in macrophage-mediated killing of *A. baumannii*. However, after 48 h incubation, both L-NAME and D-NAME treated cells showed similar level of bacterial inhibition (data not shown). Treatment of J774 cells with L-NMMA, another NO inhibitor, for 24 h also showed a similar reduction in the killing of phagocytosed *A. baumannii* as L-NAME (Fig. 8). These results indicate that NO plays a moderate role in the killing of phagocytosed *A. baumannii* by macrophages in the first 24 h after infection. Thus, it is likely that NO contributes to the bactericidal function of macrophages at an early stage of infection before sufficient numbers of neutrophils are recruited at the site of infection. At the later stage of infection, the contribution of NO could be largely compensated or replaced by other more efficient mechanisms (such as neutrophil-mediated killing). These results are in agreement with our previous *in vivo* work, which showed that iNOS knockout mice are only slightly more susceptible to i.n. *A. baumannii* infection than wild-type mice [9].

**Figure 9:**
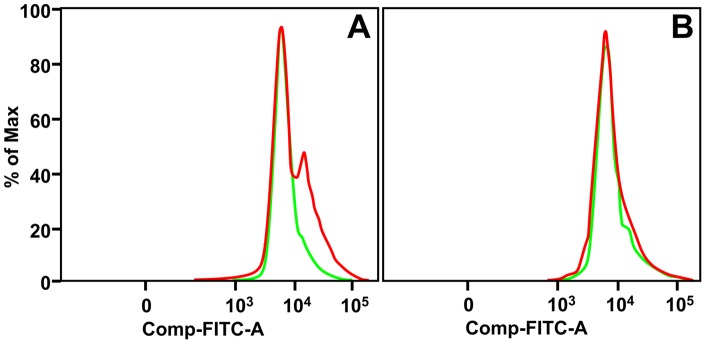
ROS production by J774 macrophages. Cultured J770 cells were incubated with CM-H_2_DFDA and stimulated with PMA (A) for 30 min or *A. baumannii* (B) for 2 h. ROS production was measured by FACS analysis of the change in fluorescence intensity upon the treatment. Green line: control treatment; red line: PMA (A) or *A. baumannii* (B) treatment, respectively.

Since our previous studies have shown that NADPH oxidase-deficient (gp91^phox−/−^) mice that lack the production of reactive oxygen species (ROS) are highly susceptible to *A. baumannii* infection [9], we also measured ROS production by J774 macrophages in response to *A. baumannii* infection, and its potential contribution in the subsequent killing of *A. baumannii*. In contrast to NO responses, we found that J774 cells, either at rest or infected with *A. baumannii*, produced virtually no ROS although its production increased substantially after 1 h phorbol 12-myristate 13-acetate (PMA) stimulation (Fig. 9). Although our *in vitro* study excluded any significant contribution of ROS in macrophage-mediated killing of *A. baumannii*, it remains possible that *in vivo*, the macrophages may be activated by a matrix of cytokines and inflammatory mediators and produce ROS to eliminate phagocytosed *A. baumannii*. Further studies of the activation of macrophages by cytokines and other mediators during *A. baumannii* infection are necessary to resolve this question.

### Bactericidal effect of NO on *A. baumannii*

**Figure 10:**
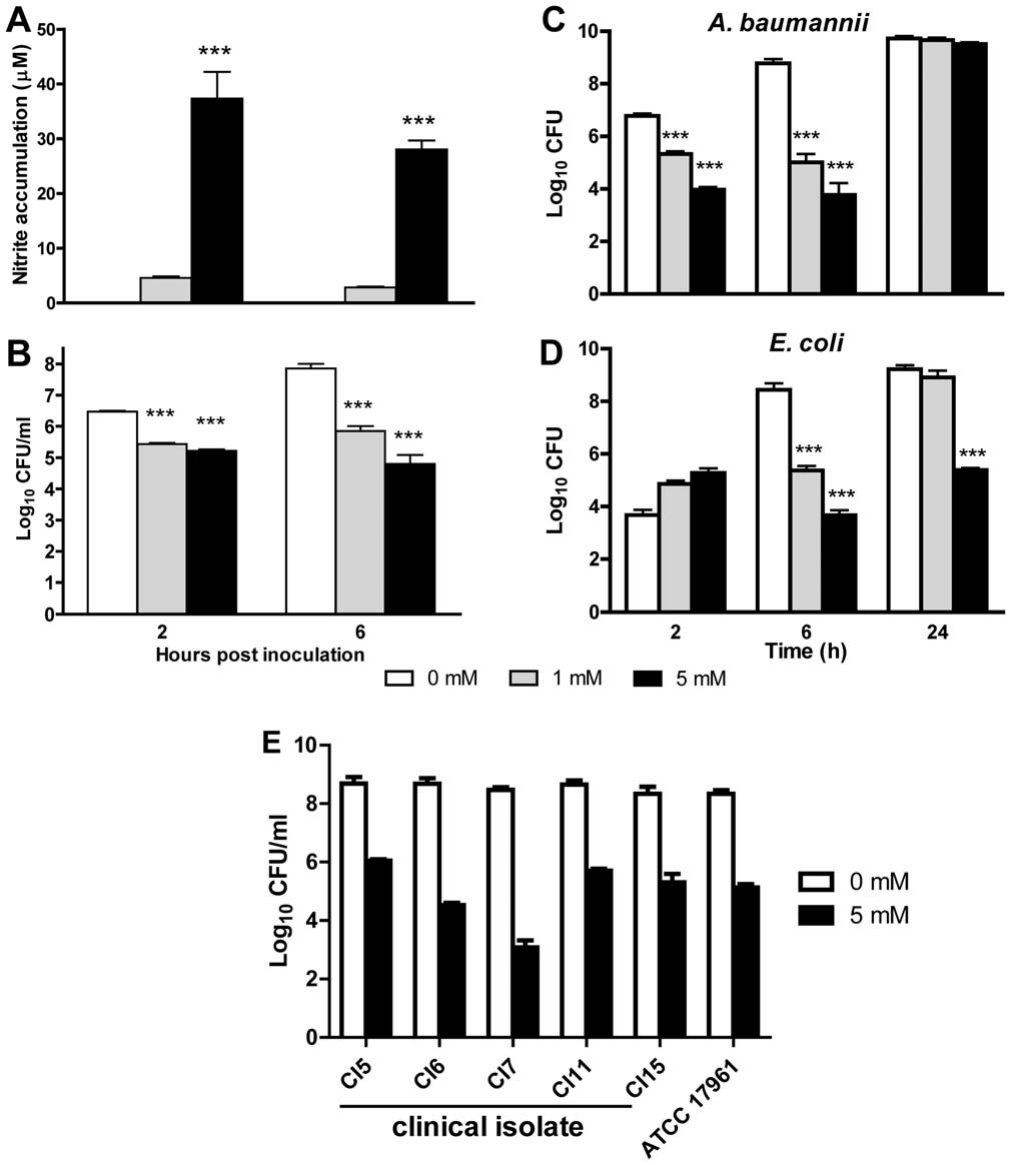
The effect of the NO-donor DETA/NO on viability of *A. baumannii* grown in broth culture. 10^5^ CFU freshly grown bacteria were treated with 0, 1.0 or 5.0 mM of DETA/NO for 2, 6 and 24 h. (A) The nitrite concentration in the culture broth at 2 and 6 h after DETA/NO addition in two of the three experiments. (B) The numbers of viable bacteria at 2 and 6 h were determined by quantitative bacteriology. The data are representative of 3 independent experiments. ***P<0.001 vs 0 mM. (C and D) Comparative susceptibility of 10^5^ CFU *A. baumannii* (C) or *E. coli* (D) upon exposure to 0, 1.0 or 5.0 mM of DETA/NO for 2, 6 and 24 h. ***P<0.001 vs 0 mM. (E) Comparative susceptibility of exposure of various *A. baumannii* clinical isolates and the ATCC 17961 strain to 5 mM DETA/NO for 6 h.

The above results (Fig. 8) suggest that NO only partially contributes to the killing of *A. baumannii* by J774 macrophages, and *A. baumannii* is only moderately susceptible to the bactericidal effect of NO. We next tested the susceptibility of *A. baumannii* to NO directly in bacterial culture broth supplemented with an NO donor, DETA/NO which induced NO production in the culture broth (Fig. 10A). The viability of *A. baumannii* in the broth supplemented with 1 or 5 mM NO donor was reduced by approximately 1 log after 2 h, and by approximately 2 logs and 3 logs respectively after 6 h incubation (P<0.001)(Fig. 10B). By 24 h after DETA/NO addition, *A. baumannii* viability is similar at all concentrations of DETA/NO (Fig. 10C) while viability is still significantly reduced in the more susceptible *E. coli* bacteria (P<0.01) at the 5 mM dose of DETA/NO (Fig. 10D). In addition, we also compared the susceptibility of five different clinical isolates of *A. baumannii* with strain ATCC 17961 to 5 mM DETA/NO and found that all of these isolates had similar susceptibilities (Fig. 10E). In contrast, more than 3 log reduction in *A. baumannii* was observed after only 1 h incubation in the presence of 5 mM H_2_O_2_ (data not shown). These data confirm that *A. baumannii* is only moderately susceptible to the bactericidal effect of NO.

### Alveolar macrophages function in host resistance to intranasal *A. baumannii* infection in mice

**Figure 11:**
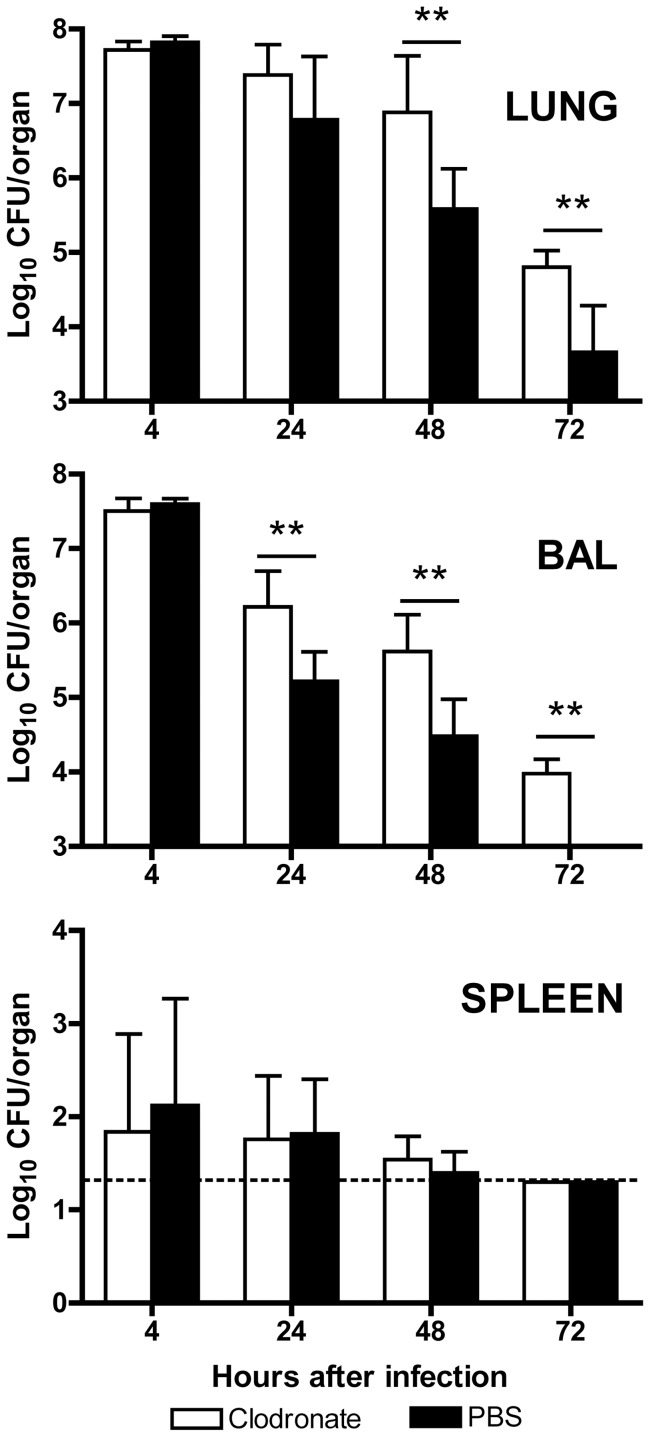
*In vivo* depletion of alveolar macrophages enhances the host susceptibility to i.n. *A. baumannii* challenge. Groups of C57BL/6 mice were i.n treated with either clodronate-liposomes to deplete alveolar macrophages or PBS-liposomes as treatment controls. Twenty-four hours later, all mice were i.n infected with 1×10^8^ CFU of freshly cultured *A. baumannii*. Five mice from each group were euthanized at 4, 24, 48 and 72 h after the infection. The bacterial burdens in the lung, BAL fluid, and spleen were determined by quantitative bacterial culture. Dashed line indicates lower limit of detection (1.3 log_10_). Data are presented as mean ± SD (n = 5). **P<0.01.

**Figure 12:**
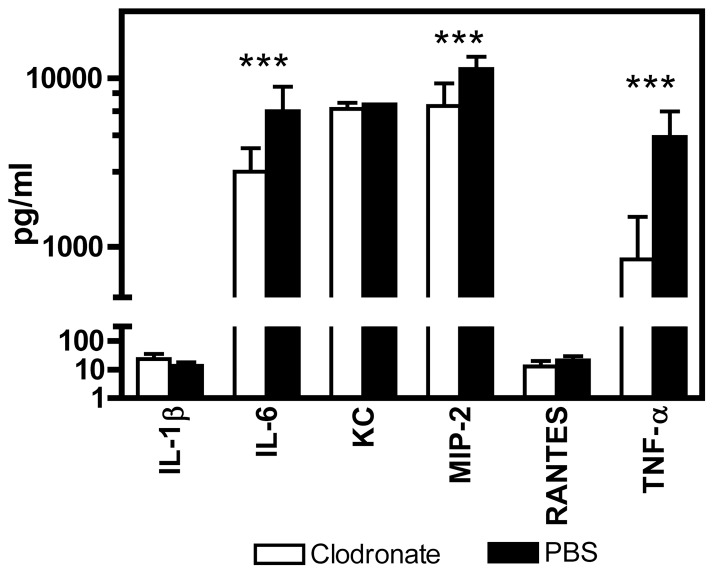
Depletion of alveolar macrophages alters early proinflammatory cytokine and chemokine responses to i.n. *A. baumannii* challenge. Groups of C57BL/6 mice were i.n. treated with either clodronate-liposomes to deplete alveolar macrophages or PBS-liposomes as treatment controls. Twenty-four hours later, all mice were i.n. infected with 1×10^8^ CFU freshly cultured *A. baumannii*. The mice were euthanized at 4 h after the infection and their lungs were lavaged. The concentrations of indicated cytokines/chemokines in the BAL fluid were measured using the mouse panel of Fluorokine MAP Multiplex kits on a Luminex 100 IS system. The detection limit for all cytokines and chemokines is <10 pg/ml. Data are presented as means ± SD (n = 5). ***P<0.01 vs. PBS-treated mice.

Results of the above *in vitro* experiments (Figs. 4, 5, 6, 7, 8, 9, 10) suggest that macrophages may play some role in the early stage of host defense against *A. baumannii* infection. We next examined the role of AMs in host defense against i.n. *A. baumannii* infection in AM-depleted mice. Groups of C57BL/6 mice were intranasally treated with clodronate-liposome to deplete AMs (AM-depleted mice) or phosphate buffered saline (PBS)-liposome as treatment control (control mice) [15], [27]. Twenty-four hours later, the mice were i.n. inoculated with 1×10^8^ CFU *A. baumannii*, and bacterial burdens in the lung, BAL fluid and spleen were determined at 4, 24, 48 and 72 h after infection (Fig. 11). The bacterial burdens in the lung and BAL fluid were not significantly different between AM-depleted and control mice at 4 hpi, but at 24 hpi significantly higher numbers of bacteria were recovered in the BAL fluid of AM-depleted mice (P<0.01). At 48 and 72 hpi, significantly more bacteria were detected in both the lung and BAL fluid of AM-depleted mice (P<0.01). The overall bacterial burdens in the spleens of infected mice were relatively low, and there was no significant difference between AM-depleted and control mice at any time point. We also determined a panel of 6 proinflammatory cytokine and chemokine levels in the BAL fluid of these mice at 4 hpi and found that the AM-depleted mice had significantly lower levels of IL-6, MIP-2 and TNF-α (p<0.001) than control mice (Fig. 12), whereas the levels of IL-1β, KC, and RANTES were similar between the two groups of mice.

These results indicated that AMs do play a subtle, but important role in early host resistance against i.n. *A. baumannii* infection. However, in comparison with previous studies with neutrophil-depleted mice [7], [11], the contribution of AMs to overall host defense in this model is relatively insignificant. Depletion of neutrophils converted a self-limiting infection into a lethal infection, whereas there was no death observed in AM-depleted mice in this study. In addition, the bacterial burdens in the lungs of neutropenic mice are about 100 times higher than control IgG-treated mice [7], while the difference between AM-depleted and control mice was only about 10 times (Fig. 11). Several factors may contribute to the different resistance observed between AM-depleted and neutropenic mice during i.n. *A. baumannii* infection. Firstly, AMs may be inherently less efficient in killing *A. baumannii* since our *in vitro* experiments showed that it required 24 h for AMs to kill 80–90% of phagocytosed *A. baumannii* while neutrophils can kill a similar percentage of bacteria in one hour (Fig. 7). Secondly, clodronate-liposome administration can only deplete about 80% of AMs and the depletion is largely restricted to terminal bronchioles and alveolar space [28]. On the other hand, RB6-8C5 monoclonal antibody treatment depletes >90% of pulmonary neutrophils and >95% of circulating neutrophils for at least 2 days [7]. Finally, although administration of clodronate-liposome depleted AMs, it is likely that this also induced the recruitment of a small number of neutrophils, presumably in response to the cellular debris and soluble molecules released from the lysed macrophages. These neutrophils may partially compensate for the loss of AMs in control of bacterial replication. Nevertheless, the early phagocytosis and killing of bacteria by AMs may be important to contain the initial bacterial burden until neutrophils can be recruited to kill *A. baumannii* more efficiently and clear the infection.

**Figure 13:**
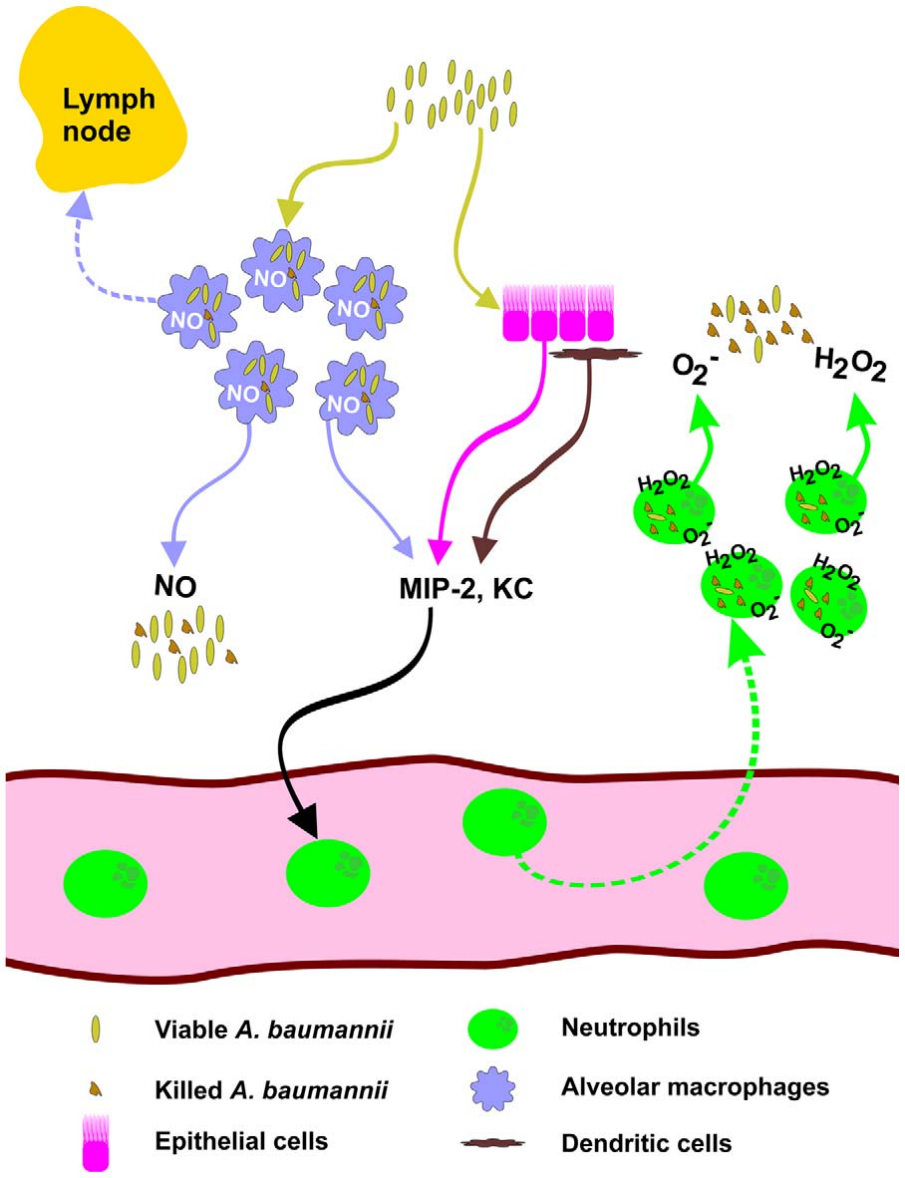
Schematic depiction of the potential role of alveolar macrophages during early host defense against intranasal *A. baumannii* infection. Intranasally inoculated *A. baumannii* are rapidly taken up by resident alveolar macrophages (AM) shortly after infection. The infection stimulates the synthesis and production of bactericidal molecules such as nitric oxide (NO) which kills some *A. baumannii* inside AMs or in the alveolar space and partially controls the replication of *A. baumannii*. Some AMs containing viable *A. baumannii* migrate to the regional lymph nodes and initiate extrapulmonary dissemination. The proinflammatory cytokines/chemokines (such as MIP-2 and KC) produced by AMs and other pulmonary resident cells (such as dendritic cells and alveolar epithelial cells) in response to *A. baumannii* induce the recruitment of neutrophils from peripheral circulation into bronchoalveolar spaces where they release oxidative radicals (O_2_^−^ and H_2_O_2_) to clear the infection.

In summary, the interaction between *A. baumannii* and the host innate immune system is likely to govern the extent of bacterial proliferation and local host inflammatory response following pulmonary bacterial infection. In this regard, AMs are the predominant phagocytes in the lung, and reside in the distal airway and alveolar spaces. They form the front line of phagocytes that initiate early innate immune responses against pulmonary pathogen invasion, as schematically summarized (Fig. 13). In this regard, macrophages express a wide array of pathogen pattern recognition molecules (such as TLRs, surface scavenger receptors and mannose receptors) to recognize and phagocytose bacterial and non-bacterial pathogens, and initiate inflammatory responses [29]–[30]. In this study, we used both an *in vitro* macrophage cell line and an AM depletion mouse model to examine the function of macrophages in the phagocytosis, killing, and regulation of inflammatory responses during *A. baumannii* infection. Our results showed, for the first time, that although macrophages play a relatively minor role in the overall host defense against *A. baumannii* infection, they play an important role in the initial stage of host defense against respiratory *A. baumannii* infection partially through an NO-dependent mechanism. Although at this stage it is not clear whether macrophage dysfunction directly contributes to any nosocomial or community-acquired *A. baumannii* infection, many studies have revealed that some risk factors (such as cigarette smoke or alcohol abuse) are positively associated with nosocomial and community-acquired infections including *A. baumannii* [25], [31]–[33]. Smoke or alcohol consumption is also correlated with impaired immune responses including AM dysfunction in phagocytosis, killing of bacteria, and cytokine secretion [25], [34]–[37]. Further study of these risk factors and the correlated macrophage dysfunction may provide more detailed information about nosocomial and community-acquired *A. baumannii* infection.

## Materials and Methods

### Mice and cell lines

Eight- to twelve-week-old C57BL/6 mice were purchased from Charles River Laboratories (St Constant, QC, Canada). They were housed under specific-pathogen-free conditions in the Animal Resources, Institute for Biological Sciences, National Research Council Canada (Ottawa) and given free access to sterile water and certified mouse chow. The animals were maintained and used in accordance with the recommendations of the Canadian Council on Animal Care Guide to the Care and Use of Experimental Animals. This study and all animal care/use protocols were approved (ID # 2006.20, 2009.12 and 2010.18) by the Institute for Biological Sciences (National Research Council Canada) Animal Care Committee.

The mouse macrophage-like cell line J774A.1 (ATCC TIB-67, J774) was obtained from American Type Culture Collection (Manassas, VA). J774 is a well-established mouse macrophage-like cell line that has been commonly used in functional studies of phagocytosis and killing because of its similarity to normal macrophages [23]. All cells were maintained in Dulbecco's Modified Eagle Medium (DMEM) (Sigma-Aldrich, St. Louis, MO) supplemented with 10% heat-inactivated fetal bovine serum and 10 mM HEPES (Invitrogen, Burlington, ON) at 37°C with 5% CO_2_.

### Intranasal infection of mice with *A. baumannii*

For each *in vivo* experiment, fresh inocula were prepared as previously described [7]. Anesthetized mice were inoculated i.n. with ∼10^8^ CFU *A. baumannii* in 50 μl saline. This dosage induces a self-limiting bronchopneumonia in C57BL/6 mice without mortality [7]. Actual inoculum concentrations were determined by plating 10-fold serial dilutions on brain-heart infusion agar supplemented with 50 μg/ml streptomycin (BHI-S). Mice were euthanized at the indicated times and the lungs were lavaged five times with a total of 5.0 ml PBS supplemented with 3 mM EDTA as previously described [7], [38]. The total number of BAL cells was determined with a hemacytometer and differential cell counts were determined by examining 200 cells on cytospin slides (Cytospin 3, Shandon, Pittsburgh, PA) stained with Hema-3® (Fisher Scientific, Kalamazoo, MI)[7].

### FACS analysis of BAL cells

The percentage and activation of AMs in the BAL fluid were determined by FACS analysis as described previously [39]. Briefly, BAL cell samples were washed in PBS containing 1% BSA. The cells were incubated with unlabeled anti-CD16/CD32 (clone 2.4G2) monoclonal antibodies (BD Biosciences) for 15 min to block non-specific Fc receptor binding. Aliquots containing ∼10^6^ cells were stained with antibody cocktails containing appropriate fluorochrome-conjugated mAb for 30 min at 4°C. The anti-CD11c (clone HL3) mAb was used as a marker for AMs and the activation of CD11c+ AMs was further analyzed using anti-CD80 (clone 16-10A1), anti-CD86 (clone GL1), anti-CD40 (clone 3/23), and anti-MHCII (AF6-120.1) antibodies. After staining, the cells were washed twice with the above PBS/BSA solution, fixed with 200 μl of 1% paraformaldehyde (Polysciences Inc., Warrington, PA), and stored in the dark at 4°C until ready for quantitative analysis. The data were acquired using a FACS Canto flow cytometer (BD Biosciences, San Jose, CA) and analyzed using FlowJo software (Tree Star, Inc., Ashland, OR).

### *In vitro* infection of J774 cells with *A. baumannii*

Freshly grown *A. baumannii* (ATCC 17961) were prepared from a frozen stock as previously described [7]. The bacterial cells were then concentrated in Tryptic Soy Broth (TSB) with 40% glycerol and aliquots were stored at −80^°^C for use in infection studies. One ml aliquots of J774 (5×10^5^ cells/ml) cells were seeded into 24-well tissue culture plates (Becton Dickinson, Mississauga ON). After overnight culture, confluent cells were infected with 100 MOI of *A. baumannii* in 500 μl complete medium per well for 2 h as described by Choi et al.[40]. The cells were then washed 3x with HBSS and incubated for another 2 h in fresh complete medium containing 100 μg/ml gentamicin to kill extracellular bacteria.

### Phagocytosis assay

J774 cells that were incubated with *A. baumannii* for the indicated times were lysed with 0.1% Triton X-100 after a 2-h incubation with gentamicin. The number of viable bacteria recovered was determined by culturing serial dilutions of the lysates on BHI-S plates. The phagocytosis index was calculated by using the following formula: [bacteria recovered (CFU/ml)/bacteria inoculated (CFU/ml)]×100 = % phagocytosed. The same phagocytosis assay was used to test time-dependent phagocytosis and the potential mechanism of *A. baumannii* phagocytosis. In these experiments, J774 cells were either co-incubated with *A. baumannii* for a series of time periods ranging from 10 min to 4 h as indicated, or were treated with cytochalasin D (0.1 to 1 mg/ml), nocodazole (0.1 to 5 μg/ml), or tunicamycin (2 to 5 μg/ml)(all from Sigma-Aldrich) for 30 (cytochalasin D) or 60 (nocodazole and tunicamycin) min before the addition of *A. baumannii*. The total number of viable *A. baumannii* recovered was similarly determined and used for the calculation of phagocytosis index. Pilot experiments confirmed that all the chemical reagents showed no direct effect on the replication of *A. baumannii* or the viability of J774 cells in the dose and time used (data not shown).

### Bactericidal assay

To determine the killing of *A. baumannii* by J774 cells, the above phagocytosis assay was modified by further incubation of the infected monolayers for up to 48 h. Intracellular viable bacteria at 4, 24 and 48 h were determined as described above. For the 24 and 48 h cultures, cells were maintained in DMEM supplemented with only 10 μg/ml gentamicin to minimize the potential intracellular accumulation of antibiotics and subsequent killing of intracellular bacteria. Pilot experiments confirmed that this concentration of gentamicin completely killed the extracellular bacteria after overnight incubation (data not shown).

To study the role of nitric oxide (NO) in the killing of phagocytosed *A. baumannii* by macrophages, J774 cells were treated with L-NMMA (1 mM), or L-NAME (2 mM), or D-NAME (2 mM) 1 h before infection [41]. Cells were then infected with 100 MOI *A. baumannii* as described above. NOS inhibitors and controls were added again after infection. Intracellular viable bacteria were determined at 4, 24 and 48 h after the addition of *A. baumannii*.

### Production of cytokines, chemokines and NO by *A. baumannii*-stimulated macrophages

In some experiments, culture supernatants were harvested at 0, 4, 24 and 48 h after *A. baumannii* infection. Supernatants from sham-infected cells were also harvested at the same time points and served as baseline controls. The supernatants were filter sterilized and stored at −20°C until assay. The level of selected cytokines and chemokines were determined by using the mouse panel of Fluorokine MAP Multiplex Kits (R & D Systems, Inc. Minneapolis, MN) on a Luminex® 100 IS system (Luminex, Austin, TX). The analysis was done in duplicate, and cytokine/chemokines concentrations were calculated against the standards using Beadview® software (ver 1.03, Upstate) as described previously [7]. The NO_2_^−^ levels in the culture supernatants were determined by Griess assay [42]. Briefly, 100 μl culture supernatants were mixed with an equal volume of Griess reagent (0.5% sulfanilamide and 0.05% N-1-naphthylethylenediamide hydrochloride in 2.5% acetic acid). After 30 min incubation at room temperature, the mixture was measured spectrophotometrically at 550 nm. The NO_2_^−^ concentration was determined from a standard curve prepared with NaNO_2_.

### DETA/NO and bacterial viability

To test the direct bactericidal effect of NO, NO was generated using diethylenetriamine/nitric oxide adduct (DETA/NO, Sigma-Aldrich, St. Louis), a compound that decomposes spontaneously with a half-life of approximately 20 h [43]. Bacteria were freshly grown in TSB broth and then diluted in tryptic soy broth to a concentration of approximately 10^5^ CFU/ml and exposed to 0, 1 or 5 mM DETA/NO. The viable bacterial counts (CFU/ml) at various hours after incubation at 37^°^C were determined by plating serial dilutions on BHI plates.

### *In vivo* depletion of AMs

Alveolar macrophages were depleted by i.n. administration of liposome encapsulated dichloromethylene diphosphonate (clodronate, or CL_2_MDP, a gift of Roche Diagnostics GmbH, Mannheim, Germany). Liposomes containing clodronate (clodronate-liposomes) and liposomes encapsulating PBS (PBS-liposomes) were prepared as described previously [28]. Groups of five C57BL/6 mice were administrated with 100 µl of clodronate-liposomes or PBS-liposomes by intranasal route. Twenty-four hours later, the mice were inoculated i.n. with ∼10^8^ CFU *A. baumannii* as described above. Infected mice were sacrificed at 4, 24, 48 or 72 hpi, and the lungs, BAL fluid, and spleens were aseptically removed or collected, and used for quantitative bacteriology [7].

### Statistical analysis

Data are presented as means ± SD for each group, unless otherwise specified. Differences in quantitative measurements were assessed by Student's *t* test, one-way analysis of variance (ANOVA) followed by Dunnett's test or two-way ANOVA followed by Bonferroni's *post-hoc* multiple comparison tests, when appropriate. Differences were considered significant when P<0.05. All statistical analysis were performed using GraphPad Prism 4 (Graphpad Software, La Jolla, CA, USA).
